# Aesthetic Attitude Based on Kant’s Aesthetics of Caring Relationships in Nursing

**DOI:** 10.3390/healthcare11162324

**Published:** 2023-08-17

**Authors:** Byunghye Kong, Younjae Oh

**Affiliations:** 1 Department of Nursing, College of Medicine, Chosun University, Pilmun-Daero 309, Dong-Gu, Gwangju 61452, Jeollanam-do, Republic of Korea; bhgong@chosun.ac.kr; 2 College of Nursing, Research Institute of Nursing Science, Hallym University, Hallymdaehakgil 1, Chuncheon 24252, Gangwon-do, Republic of Korea

**Keywords:** Kant, aesthetics, caring, nurse, nursing

## Abstract

(1) Background: Although aesthetic attitude has been comprehended as one of the fundamental traits in nursing, there is a lack of discussion considering Kant’s aesthetics of caring relationships. The purpose of this study was to illuminate aesthetic and moral characteristics of caring expressed in the caring relationship between a nurse and patient and suggest a new perspective of aesthetic attitude based on Kant’s aesthetics of care ethics. (2) Methods: A theoretical reflection was contemplated regarding notions of aesthetic attitude in the caring relationship between a nurse and patient. (3) Results: human faculty of reflective aesthetic judgment to feel the beautiful and the sublime through imagination and free play in Kant’s aesthetics could be applied to the aesthetic attitude in the field of nursing. (4) Conclusions: A nurse who has trained with this aesthetic attitude can act as a moral agent and contribute to the protection and promotion of human dignity in a caring relationship.

## 1. Introduction

Nursing scholars consider caring to be a key concept in understanding what is involved in nursing and believe that it is a major issue in nursing ethics. Caring has been regarded as a fundamental moral value or a moral ideal of nursing for the protection, preservation, and improvement in human dignity over the last few decades [1,2,3]. In the nursing literature, characteristics of caring have been discussed and explored as a moral practice in virtue ethics rather than in principles-based ethics [4,5,6]. Nurses who have the virtue that includes emotional understanding as a characteristic can exhibit appropriate feelings toward a patient genuinely [7,8,9]. Notably, their altruistic emotion to help others is regarded as an aesthetic characteristic which is as essential as a moral characteristic in a caring relationship between a nurse and patient [10,11], although the concept of aesthetic characteristic is vaguely discussed in the field of nursing.

The aesthetic characteristic of caring as an art is emphasized in the description of what caring is in the previous literature on nursing ethics. The art of caring is based on the empathetic capacity to feel expression and transmit through imagination [12]. It includes aesthetic attitude in the caring relationships of nursing practice [10,13]. Several nursing ethicists have discussed the relationship between morality and art in nursing practice and called it a moral art or practical art [14,15]. They assert that the ultimate and ideal goal of nursing is to protect and enhance human dignity based on scientific and artistic knowledge. In other words, artistic nursing needs progress in the context of morality.

To achieve moral art, what aesthetic traits and dispositions should nurses develop? Our answer to this question is based on Kant’s perspectives regarding aesthetics, which define reflective aesthetic judgment as the capacity to reflectively judge an object through feeling rather than logical reasoning [16]. His idea was emphasized by Schiller, who advanced aesthetic education with the idea of impulse amusement from Kantian concepts of imagination and free play [17,18]. Aesthetic judgment is considered and applied to practical settings in various disciplines such as sociology [19] and medicine [20].

In recent empirical nursing research, the perception of aesthetic care among nurses was explored, which contributes to illuminating the ways to implement aesthetic attitudes in care [21,22]. However, it still has limitations in epistemologically explaining the meaning of aesthetic attitudes in nursing. While Carper asserted the significance of aesthetics in the advancement of nursing knowledge, such a viewpoint has been challenged among subsequent nursing scholars interested in exploring the role of aesthetics in nursing in recent decades [15,23,24,25,26]. The major critique is that Carper’s approach and descriptions of aesthetics as knowledge are insufficient. Accordingly, nursing scholars have discussed aesthetic aspects of nursing from diverse perspectives; in the recent theoretical literature, Herholdt-Lomholdt [26] argued that ontological reflection, along with epistemological understanding, is necessary to comprehend aesthetic aspects of excellence in nursing. Pols [27], based on empirical ethics, argued for a close relationship between aesthetic values and good care in nursing. Thus, the values of aesthetic nursing must be linked to a broader meaning and epistemologically comprehended based on various metaphysical perspectives.

Nevertheless, there is little literature on nursing ethics to elucidate aesthetic attitude based on Kant’s theory of aesthetics in a caring relationship between nurse and patient, although aesthetic attitude through feelings in the personal relationship between nurse and patient is deliberated as a crucial moral trait for a nurse. Thus, the purpose of this study was to illuminate aesthetic and moral characteristics of caring expressed in the caring relationship between nurse and patient and suggest a new perspective of aesthetic attitude in a caring relationship in care ethics for nurses, namely through aesthetical reflective judgment and the sublime described in Kant’s aesthetics.

## 2. Materials and Methods

A theoretical reflection was contemplated regarding notions of aesthetic attitude in the caring relationship between nurse and patient. According to Yan’s guide for theoretical articles [28], first, prior to applying Kant’s ideas on aesthetic judgment to the caring relationship, we analyzed aesthetic and moral characteristics in nursing based on nursing scholars’ perspectives on aesthetic aspects in nursing. Second, we identified the applicability of the aesthetic attitudes based on Kant’s notions of reflective aesthetic judgment and the sublime in establishing the caring relationship between a patient and nurse. Finally, we explored and suggested how aesthetic attitudes can make nursing care a beautiful virtue using Kant’s ideas on the sublime.

Concerning the fundamental theoretical base, Kant affirmed that aesthetics can be defined as the science that studies and investigates the systematic origin of pure feeling and its expression, art [11]. Kant’s notions about aesthetic attitudes were applied, which were based primarily on his influential book, the “Critique of Aesthetic Judgment” [16]. In his book, aesthetic attitudes are validated by reflective aesthetic judgment, which is a faculty to judge an object reflectively by feeling it as pleasure. This feeling does not solely convey needs and emotions; it is based on a specific type of judgment that Kant calls “reflective aesthetic judgment”. The reflective feeling arises from the free play of human imagination with understanding as cognitive powers extended to a moral idea on universal humanity.

## 3. Results

### 3.1. Aesthetic and Moral Characteristics in Nursing

Before discussing Kant’s aesthetics, this paper first discusses Watson’s claim: “nursing is a human art as well as a moral ideal” [3]. Her appreciation of “caring as art” clarifies the aesthetic and moral characteristics of caring. Although how nursing can be an art remains controversial, this paper supports Watson’s claim because she applies art as a moral expression to transpersonal caring. According to Watson, transpersonal caring is the art of caring. It is based on the capacity for compassion that one can feel, express and transmit through intuition and imagination. This art of caring comes to the moral ideal of nursing, which preserves and enhances human dignity and wholeness by pursuing a harmony of the body, mind, and soul. She notes that the art of transpersonal caring is a moral ideal of nursing that evokes, transmits, and expresses the universal feeling of humanity [3]. This caring occurs when a nurse has a feeling caused by an aesthetic attitude toward the patient. An aesthetic attitude includes an empathetic awareness by means of extended imagination that could help nurses appreciate a patient’s vulnerability and response to this in the caring relationship with them.

Imagination is essential for nurses to fully understand the unique situation that a patient suffers. That is, a nurse needs to respond sensitively to a patient’s appeal through personal emotional interpretation and moral imagination in the caring process of human-to-human interaction [8]. In enhancing these sensitive responses, Norvedt [8] asserted that epistemological knowledge and ontological essence in nursing are fundamentally involved in being-for-the-other, which is responsible for the other by responding to a human being’s suffering and vulnerability, namely ethics. Human responsibility comes from the patient’s appeal and arises from a response to the patient’s moral reality.

Human responsibility is also called ethical responsibility by Martinsen [29], who has focused on an aesthetic–artistic approach in caring. Through an aesthetic attitude, nurses can discover and experience values that are inherent in life itself, such as hope, trust, dignity, and compassion in the context of an ethical meeting. An ethical meeting emerges when the nurse becomes aware of the relationship between the nurse and patient. There, the nurse feels an ethical responsibility arising from the patient’s demand. Martinsen [29] emphasized that the concept of withdrawal is being open to emotion, not passivity, in an aesthetic–artistic approach. It treats a patient’s demands with care and respect. The withdrawal allows a nurse to thoroughly understand another person’s world by breaking her or his prior theoretical knowledge. In this approach, the meaning of aesthetics includes “perceive” or “sense”. Such senses depend on individual nurses who decide how to sense the patient.

As discussed above, nursing scholars have offered theoretical reflections regarding aesthetical traits in nurses caring for others as moral agents. It is necessary to comprehend aesthetic attitude and its moral characteristic through feelings that can form a caring relationship in nursing practice. This paper will suggest a new perspective on caring relationships in care ethics in terms of Kant’s aesthetics. It will be described in the following sections of the article.

### 3.2. Aesthetic Attitude Based on Kantian Care in Nursing

In the “Critique of Aesthetic Judgment”, Kant [16] analyzed how aesthetic attitudes are possible and how they can be distinguished from other attitudes, namely immediate affective attitude, scientific attitude, and practical–pragmatic attitudes. In his book, aesthetic attitudes are validated by reflective aesthetic judgment. It is a faculty to judge an object reflectively by feeling it as pleasure. This feeling is not merely to express needs and emotions. It is based on a certain judgment that Kant refers to as “reflective aesthetic judgment”. The feeling of pleasure in a beautiful object is felt in virtue of an exercise of reflective judgment. That kind of reflective feeling is caused by the free play of human imagination with understanding as cognitive powers extended to a moral idea on universal humanity. In other words, we feel an object is beautiful, free from the affection for the object’s existence and free from the pragmatic interest in it, which asserts its utility. Kant describes such aesthetic attitudes as disinterested pleasure (das interesslose Wohlgefallen). This feeling of pleasure is to facilitate the feeling of life through the free play of imagination with understanding, such as cognitive powers in a harmonious relationship with the object [16].

Kant suggests that aesthetic judgments appear from the free play of human imagination with understanding. The judgment has universal validity and universal communicability because all human beings have taste as a kind of common sense [16]. The notion of common sense by Kant is to be understood in terms of three maxims of thought: think for one, put oneself in thought in the place of everyone else and think consistently in agreement with one [16]. Consequently, common sense is an ability to enlarge the thinking for the idea of universal human dignity through the free play of imagination with understanding and the ability to communicate with each other through feeling. Kant says: “It indicates a man with a broadened way of thinking if he overrides private or subjective conditions of his judgment, into which so many others are locked, as it were and reflected on his own judgment from a universal standpoint of other” [16].

Arendt [30] explains communication in the public realm based on the theory of Kantian aesthetics in the Lectures on Kant’s political philosophy. Her theory of political judgment is based on Kant’s aesthetics rather than on his moral philosophy. According to her book, common sense can be understood as a capacity for publicity of thought through feeling which has universal communicability and can make one share one’s judgment or overcome one’s individual idiosyncrasies [30]. In this case, the communicability of feeling occurs to the extent that the imagination of pursuing the idea of universal humanity and free play with understanding is developed [31].

The aesthetic attitude based on the feeling of the beautiful, stated by Kant and Arendt, can be a new perspective of inter-subjective communication through a shared empathy for the caring relationship. A nurse with a cultivated taste who exercises it can be free from any personal interest, selfish desire, need, or pragmatic concerns. She or he can then make an authentic care relationship with a patient based on the feeling of universal humanity as a possibly moral idea. Namely, while a nurse reads a patient’s inner mind through the free play of imagination with understanding and feels empathy extended to universal humanity as common sense, she or he can have an aesthetic attitude toward the patient in the caring relationship [32].

Common sense, particularly as a beautiful virtue, can enable nurses to communicate with a patient through the feeling of love for mankind based on universal human dignity in the process of aesthetical reflective judgment. This is because the common sense of nurses must be achieved by transcending their private or subject conditions in favor of public and intersubjective ones and broadening the horizon of their thinking toward receptivity of the moral idea of universal humanity. Therefore, Kant’s reflective aesthetic judgment offers valuable ideas that aesthetic attitude formulated by empathy through the free play of imagination and universal humanity as common sense can help nurses preserve, maintain, and enhance the human dignity of patients in caring relationships.

### 3.3. The Sublime Based on Kantian Care in Nursing

In aesthetic judgments, the relationship between imagination and understanding is a harmonious one. As stated by Kant [16], “beautiful is directly attended with a feeling of furtherance of life and is thus compatible with charms and a playful imagination”. By contrast, the sublime occurs from the relationship between imagination and reason. It is based on conflict. Therefore, it is accompanied by a feeling of displeasure. This displeasure arises from the inadequacy of imagination in the aesthetic estimation of magnitude to attain its estimation by reason [16]. Conflict, disharmony, struggle, and violence are predominant features of the sublime. In the process, as we painfully experience the incapacity of our imagination, we discover or are reminded of our capacity for reason as independent from and superior to senses and nature [33]. Kant’s statement of “feeling of our possessing a pure and self-sufficient reason” is a core category of the sublime [16]. The sublime is a feeling of self-respect or self-esteem caused by getting over the feeling of displeasure from conflict in a relationship.

An encounter with an object does not always take us to the path of having a free play of imagination that gives us the feeling of life. Instead, we are opposed to it with a feeling of displeasure blocking a feeling of life. The feeling of displeasure halts our imagination when we reject the object presented because our mind is limited to imagining and understanding it. On the other hand, if we have trained and developed our morality based on the idea of human dignity, we would overcome the limitations of our sensibility and imagination. The mind is “incited to abandon sensibility and employ itself upon ideas involving moral law” [16].

While we are frustrated and depressed with the object that makes us experience our limitation of imagination, we can feel the sublime by keeping a distance from the object and finally reflect on ourselves by invoking the idea of morality from our mind [34]. Namely, we come to feel beautiful when we have our mind ready to love the object; by contrast, we come to experience the sublime when we respect ourselves as a moral subject [16]. The feeling of the sublime is the same as self-respect since when we meet a disagreeable object, it lets us turn back to our inner mind and reflect on ourselves morally, which simultaneously makes us respect the object [16].

Kant’s perspective on the sublime described above can be applied to a caring relationship between a nurse and patient. Nurses are often faced with a situation where they fail to have a feeling of life in an encounter with suffering patients in vulnerable situations [35]. Several nurses would rather withdraw their care from their patients or avoid those situations, such as leaving their positions to alleviate their emotional or moral distress [36,37]. In this case, nurses could not have any compassion or empathy with patients through their reflective imagination. Therefore, it is hard for them to have a feeling of life or love for patients. Instead, the closer the emotional distance between nurses and patients is, the higher possibility for nurses to get hurt emotionally [17]. On the other hand, if nurses keep a reflective distance, their imagination toward others can turn back to themselves and awaken their reasoning as a faculty for moral ideas. In that case, nurses not threatened by any sensibility from others will obtain moral subjectivity in their inner mind [38].

The sublime can arise when a nurse makes her/himself aware of the moral idea of human dignity in one’s mind and restores one’s morality and one’s relationship with a patient. Feelings of the sublime can occur anytime when a nurse interacts with a patient reciprocally. Nevertheless, a caring relationship with a patient through the sublime would not be possible if a nurse has not received higher moral training. The sublime is a kind of moral feeling which occurs when we get over our displeasure caused by rejecting a feeling of life and when we, as persons of dignity, finally restore our relationships with others.

In the end, a feeling of the beautiful or the sublime would not be possible unless a nurse has developed morality by exercising practical reason in mind. The feeling of the beautiful, which is lovely and full of life, occurs reflectively in the process of empathy for patients. Experiencing the sublime by keeping a reflective distance from them is a feeling of respect for human dignity by finding the moral idea in one’s own mind to get over one’s sensible side. The nurse feels the sublime, especially when they are sure to confirm their moral subjectivity and the dignity of others and to care for the patient without being threatened by vulnerable sensibility from the outside [13,32].

## 4. Discussion

Our review attempts to set a new perspective of aesthetic attitude for care ethics in the relationship between nurses and patients through pure aesthetic judgments and the sublime, which constitute the main issues in Kant’s aesthetics. The aesthetic attitude enables caring relationships and caring actions to maintain and protect human dignity as a moral ideal in nursing.

Our findings are aligned with previous studies on the aesthetic aspects of nursing. According to Herholdt-Lomholdt [26], the aesthetic aspects of nursing involve perceiving a shared understanding of human experiences that may lie beneath the surface or in between interactions. They can also be seen as glimpses of something beyond ordinary encounters and as a recognition of the deeper meaning embedded within the situation. In Nåden and Eriksson [39]’s qualitative studies on moral attitudes in nursing care, the helpers’ moral attitude becomes evident in two ways: firstly, by actively acknowledging and observing the patient’s suffering, where they provide support and follow the patient’s lead, and secondly, through their sense of accountability and their actions on behalf of others. Such moral values pursued by nurses are directly connected to, and essential for, the preservation of the dignity of human beings.

Our review remarked that in response to subjective and individual situations, nurses should overcome the passive sensibility that makes them vulnerable to patients. Nurses should not avoid a patient’s call. They should accept this as a primary and fundamental duty. Our finding is aligned with other studies. Gastmans [1] argued that nursing care is a moral practice in which ethical concerns for a patient’s well-being and dignity are fundamental to the moral demand that inspires nursing care. The essence of nursing care is to respond to vulnerability. Nurses are driven to meet a patient’s needs in a suitable and fitting manner when faced with various types of vulnerability. For instance, in our previous research on ethical conflicts experienced by geriatric hospital nurses who care for persons with dementia [34], nurses reported a strong sense of responsibility to care for patients when they felt the patient was placed in a situation where she or he was completely dependent. Such a strong responsibility was also considered as their ethical duty as nurses.

Also, nurses should keep a reflective distance and obtain their own autonomy to help them reflect their own morality. This notion has been practiced in maintaining therapeutic distance among nurses between patients and nurses and has contributed to applying knowledge to practice as the accomplishment of phronesis in nursing. Therapeutic distance has been emphasized in nursing education to provide standardized nursing care, in which the premise is to acknowledge personal and holistic human beings [40]. Indeed, maintaining therapeutic distance is critical in psychiatric settings to establish therapeutic relationships between patients and nurses [41,42]. That can also be explained by the notion of “withdrawal” by Martinsen [43]. Austgard [44]’s statement based on Martinsen’s concept of withdrawal is that withdrawal does not mean passivity but presupposes an aesthetic–artistic approach, referring to the ancient Greek word “aesthesis”, giving the meaning “perceive” or “sense”. To be professional is to be open to an emotion that sends a person into a state of withdrawal. The withdrawal gives the other person the opportunity to come forward and be a person of importance to the nurse [45]. According to Martinsen, various modalities can be employed by the senses, even though not all possess ethical implications. Hence, it falls upon the nurse as an individual to discern and determine the appropriate approach for perceiving the patient.

In this regard, we found that a nurse’s aesthetic attitude can have aesthetic and moral characteristics that form a caring relationship in nursing. Stikholmen et al. [46] explained aesthetics in nursing with external aesthetic dignity based on the concept of relative dignity. According to them, “The relative dignity is divided into an inner ethical dignity and an external aesthetic dignity, both of which are symbols or expressions of absolute dignity. The inner ethical dignity is a psychic dimension for the experience of dignity. It can be expressed as pride, nobility, position, rank and independence. The inner ethical dignity draws attention to one’s own and others’ dignity and contains values such as morality, ethical attitude, principles and ideals. The external aesthetic dignity is a bodily dimension that is expressed in concrete actions, attributes, products and symbols. It contains values such as respect, grandiosity, properness, suitability, restraint and orderliness, and focuses on how dignity is expressed in action”. In instances where a value pertaining to inner or external dignity becomes unattainable, thereby posing a threat to an individual’s dignity, it is plausible that an alternate value may supplant the original, symbolizing human dignity and ultimately restoring it [47]. Thus, nurses should be qualified with an aesthetic attitude based on morality for a caring relationship to protect and promote human persons’ dignity.

The sublime is particularly a feeling of moral self-esteem and respect for others. The sublime is a delight of reason. When a nurse gets over the limit of the sensible side of their being, conflict, resistance and emotional suffering with her or his patients, she or he can experience the sublime through the feeling of respect for moral law based on the idea of human dignity in the inner mind. According to Rodríguez-Prat [48]’s narrative study about experiencing the sublime in a palliative care unit, the sublime the author glimpsed was achieved through health care workers’ care driven by an authentic moral attitude, acknowledging the dignity in another person, such as being present and silent or holding patients’ hands. Meanwhile, moral self-esteem can support the argument for self-care as an ethical obligation for nurses [49]. Moreover, struggling during the COVID-19 pandemic era, nurses and citizens realized that the extent of self-protection of and self-improvement in nurses’ health can crucially influence not only patients’ but also citizens’ health and well-being. Considering Gastmans’s argument that the caring process is accomplished through a dialogical interpretative process, reciprocity can be essential to build the caring relationship and to facilitate nurses’ moral self-esteem [1]. That is, in the context of patient–nurse dynamics, it is evident that patients demonstrate a display of respect towards their nursing professionals, thereby permitting themselves to receive compassionate attention and responding suitably to the care administered.

To achieve and develop higher morality among nurses, both personal and organizational efforts are required for nursing in practical settings and in academic fields in order to pursue the sublime as a moral ideal. Empirical research can gradually reduce gaps between theory and practice and comprehend the meaning and essence of aesthetic experiences in the caring relationship more precisely. Future empirical research is required to deal with nurses’ perspectives and experiences of feelings of the beautiful and the sublime in nursing care.

## 5. Implications for Nursing Practice

Our findings can be applied to nursing education to improve nurses’ ethical competence. Aesthetic attitudes in nursing care should be more emphasized in nursing education. To improve aesthetic attitudes among nurses, most of all, empathy empowerment and understanding of human beings’ dignity through moral imagination are essential. Education for cultivating moral imagination should be stressed. Indeed, moral imagination can be improved through ethics education; it has been recently reported that constructive educational approaches using learning methodologies based on high-fidelity simulation or scenario-based role play can stimulate learners’ moral imagination [50,51]. Recently, Gerrits et al. [51] reported the positive effects of education regarding moral imagination among biomedical researchers on improving moral sensitivity and moral reasoning. Such moral imagination enables nurses to obtain common sense regarding the meanings of universal humanity and the dignity of a human person as beautiful virtues. In addition, we suggest that the feelings of the sublime require self-effort; nurses should develop such feelings individually through personal efforts such as reflective aesthetic judgment on their nursing care. For instance, nurses can exercise moral emotions like compassion or empathy through universal humanity while establishing a caring relationship by keeping a distance from the patient.

## 6. Conclusions

This paper develops an understanding of aesthetic attitudes based on Kantian concepts of caring relationships in nursing. In Kant’s aesthetics, the human faculty of aesthetic judgment to feel the beautiful and sublime has essential implications for aesthetic attitudes in performing caring actions in human relationships. This is because aesthetic judgment enables nurses to transcend their own privacy to broaden the horizon of thinking towards receptivity of the moral idea of universal humanity, named common sense, through the free play of imagination. Such an aesthetic attitude based on morality for a caring relationship enables nurses to protect and promote human persons’ dignity through common sense. The faculty of aesthetic judgment can help nurses communicate with each other through the feeling of empathy based on the moral idea. Essentially, a nurse’s feeling of empathy or love for a patient through reflective aesthetic judgment makes it possible to provide virtuous care as moral practice. The sublime can rise when a nurse makes herself/himself aware of the moral idea of human dignity in one’s mind and restores one’s morality and one’s relationship with a patient.

## Data Availability

Not applicable.
